# Supplementary material for: A systematic analysis on the clinical safety and efficacy of onco-virotherapy
Source: Mol Ther Oncolytics. 2021 Oct 5;23:239–53. doi: 10.1016/j.omto.2021.09.008 (PMC8551473; doi:10.1016/j.omto.2021.09.008)
Supplement: Document S1. Supplemental materials and methods [file mmc1.pdf]

OMTO, Volume *23*

Supplemental information

A systematic analysis on the clinical safety and efficacy of onco-virotherapy

Darshak K. Bhatt, Lieske Wekema, Luciana Rodrigues Carvalho Barros, Roger Chammas, and Toos Daemen

Supplementary Information

1. **Search Strategy**

We retrieved clinical trials from the *clinicaltrials.gov* registry (https://clinicaltrials.gov) and PubMed (http://www.ncbi.nlm.nih.gov/PubMed) and EMBASE (https://www.embase.com) databases at the start of August 2020. For each medium we used a different search strategy, as specified below.

- *ClinicalTrials.gov*

We obtained clinical trials from the *clinicaltrials.gov* registry using the following settings:

Status: “All studies” / Condition or disease: “Cancer” / Other terms: “Virus”

- *PubMed*

In cooperation with the Central Medical Library (CMB) of the University Medical Center Groningen (UMCG), we constructed a PubMed search strategy based on our PICOS framework:

```
("Neoplasms"[Mesh] OR "Oncolytic Virotherapy"[Mesh] OR neoplasia*[tiab] OR neoplasm*[tiab] OR tumor[tiab] OR tumors[tiab] OR tumour*[tiab] OR cancer*[tiab] OR malignan*[tiab] OR carcinoma*[tiab] OR metasta*[tiab] OR melanom*[tiab] OR oncolyt*[tiab])
AND
("Oncolytic Virotherapy"[Mesh] OR "Oncolytic Viruses"[Mesh] OR virotherap*[tiab] OR (oncoly*[tiab] AND (virus*[tiab] OR viral[tiab] OR adenovir*[tiab] OR reovirus*[tiab] OR poxvirus*[tiab] OR gene[tiab] OR genes[tiab] OR herpes*[tiab])) OR ((administration*[ti] OR target*[ti] OR therap*[ti] OR treatment[ti] OR intratumo*[tiab] OR inject*[ti] OR intravenous[ti] OR immunotherap*[ti]) AND (virus*[ti] OR reolysin[ti] OR reovirus*[ti] OR poxvirus[ti] OR adenovirus*[ti]))  OR replicon*[tiab])
```

AND

("Clinical Trial" [Publication Type] OR "Clinical Trials as Topic"[Mesh] OR "Cohort Studies"[Mesh] OR "Observational Study" [Publication Type] OR "Case Reports" [Publication Type] OR "Prognosis"[Mesh] OR random*[tiab] OR controlled study[tiab] OR clinical study[tiab] OR trial[tiab] OR cohort[tiab] OR prospectiv*[tiab] OR follow-up[tiab] OR study[ti])

NOT

("Animals"[Mesh] NOT "Humans"[Mesh])

NOT

(epstein[tiab] OR epstein[ti] OR EBV[tiab] OR papillomavir*[tiab] OR papillomavir*[ti] OR hepatitis*[tiab] OR hepatitis[ti] OR HCV[tiab] OR HCV[ti] OR HBV[ti] OR HBV[tiab] OR HIV[tiab] OR HIV[ti])

- *EMBASE*

In cooperation with the CMB of the UMCG, we re-wrote the PubMed search strategy into a search strategy that was suitable for the EMBASE database:

('neoplasm'/exp OR 'oncolytic virotherapy'/exp OR (neoplasia* OR neoplasm* OR tumor OR tumors OR tumour* OR cancer* OR malignan* OR carcinoma* OR metasta* OR melanom* OR oncolyt*):ab,ti)

AND

('oncolytic virotherapy'/exp OR 'oncolytic virus'/exp OR virotherap*:ab,ti OR (oncoly* AND (virus* OR viral OR adenovir* OR reovirus* OR poxvirus* OR gene OR genes OR herpes*)):ab,ti OR ((administration* OR target* OR therap* OR treatment OR intratumo*

OR inject* OR intravenous OR immunotherap* OR trial) AND (virus* OR reolysin OR reovirus* OR poxvirus OR adenovirus*)):ti OR replicon*:ab,ti)

AND

('clinical trial'/exp OR 'intervention study'/exp OR 'longitudinal study'/exp OR 'major clinical study'/exp OR 'prospective study'/exp OR 'cohort analysis'/exp OR 'follow up'/exp OR 'observational study'/exp OR (random* OR ‘controlled study’ OR ‘clinical study’ OR trial OR cohort OR prospectiv* OR ‘follow-up’):ab,ti OR study:ti)

NOT

('animal'/exp NOT 'human'/exp)

NOT

('conference abstract'/it OR 'review'/it)

NOT

(hepatitis:ti OR hiv:ti OR 'epstein barr':ti)

2. **Supplementary table 1, containing the dataset obtained after systematic search and review; with each article or trial listed in row, while respective variable of interest organized in columns.**
