# Supplementary material for: A systematic analysis on the clinical safety and efficacy of onco-virotherapy
Source: Mol Ther Oncolytics. 2021 Oct 5;23:239–53. doi: 10.1016/j.omto.2021.09.008 (PMC8551473; doi:10.1016/j.omto.2021.09.008)
Supplement: Document S2. Article plus supplemental information [file mmc3.pdf]

Molecular Therapy
Oncolytics
Original Article

A systematic analysis on the clinical safety and efficacy of onco-virotherapy

Darshak K. Bhatt,[1,2] Lieske Wekema,[1] Luciana Rodrigues Carvalho Barros,[2] Roger Chammas,[2] and Toos Daemen[1]

[1]Department of Medical Microbiology and Infection Prevention, University Medical Center Groningen, University of Groningen, 9713 AV Groningen, the Netherlands; [2]Center for Translational Research in Oncology, Instituto do Câncer do Hospital das Clínicas da Faculdade de Medicina da Universidade de São Paulo, São Paulo, CEP 01246-000, Brazil

Several onco-virotherapy candidates have been developed and clinically evaluated for the treatment of cancer, and several are approved for clinical use. In this systematic review we explored the clinical impact of onco-virotherapy compared to other cancer therapies by analyzing factors such as trial design, patient background, therapy design, delivery strategies, and study outcomes. For this purpose, we retrieved clinical studies from three platforms: ClinicalTrials.gov, PubMed, and EMBASE. We found that most studies were performed in patients with advanced and metastatic tumors, using a broad range of genetically engineered vectors and mainly administered intratumorally. Therapeutic safety was the most frequently assessed outcome, while relatively few studies focused on immunological antitumor responses. Moreover, only 59 out of 896 clinical studies were randomized controlled trials reporting comparative data. This systemic review thus reveals the need of more, and better controlled, clinical studies to increase our understanding on the application of onco-virotherapy either as a single treatment or in combination with other cancer immunotherapies.

INTRODUCTION

In the last two decades, viral vector-based therapies are gaining increasing attention as a promising strategy for cancer treatment. Studies in the field of cancer virotherapy have explored the administration of viral vectors as agents for therapeutic vaccines,[1] gene therapy,[2–4] and more recently as oncolytic therapeutics.[5,6] To date, several viral vectors are approved for clinical application. Safety and efficacy are the primary goals of clinical trials and, therefore, the clinical success of cancer virotherapy depends on these outcomes. Advanced genetic engineering tools have allowed researchers to improve safety by enhancing tumor targeting and tumor replication.[5] Additionally, with these tools, the efficacy of viral vectors for onco-virotherapy can be enhanced by encoding transgenes that strengthen the oncolytic potential or that elicit stronger antitumoral immune responses.[7] Besides vector design, factors such as clinical trial design, patient background, dose, frequency, delivery strategy, issues related to immune-mediated virus elimination, and the choice of clinical outcome measures may also influence clinical success. In this study we aimed to analyze these parameters based on a systematic review. For this purpose we retrieved articles from several platforms in the context of onco-virotherapy for antitumor responses. Previous systematic reviews on onco-virotherapy were based on a limited number of articles retrieved exclusively from PubMed,[6,8] thereby overlooking studies archived by other platforms, while a recent review focused on randomized controlled trials only.[9] To provide a broader overview of the global trends in clinical research related to onco-virotherapy we conducted an extensive literature survey that includes a more complete set of articles and trials retrieved from multiple platforms, including ClinicalTrials.gov, PubMed (Medline), and EMBASE. For a comprehensive analysis of the clinical research in onco-virotherapy to date, our dataset includes phase I–IV trials, along with cohort and case studies.

RESULTS

Scenario of clinical studies evaluating onco-virotherapy

A systematic search performed on PubMed, EMBASE, and ClinicalTrials.gov, retrieved until August 2020, found 249 trials, 331 articles on PubMed, and 316 articles on EMBASE that contained relevant terms (Supplemental information) and fulfilled the necessary inclusion criteria required for the analysis on clinical data related to onco-virotherapy (Figure 1A). Of these trials and articles, 59 entries contained data from controlled clinical trials, allowing the comparison of onco-virotherapy with either placebo, standard palliative care, or conventional therapy (Figure 1A). Most of these studies were performed in North America, the Republic of China, and Europe (Figure 1B).

There has been an increase in the number of studies in the past two decades, which can be attributed to the widespread availability of genetic engineering platforms and molecular techniques to design and test onco-virotherapy in both pre-clinical and clinical stages (Figure 2A). Especially the approval of talimogene laherparepvec[10] (T-VEC, herpes virus with infected cell protein [ICP] 34.5 and ICP47 deletion, encoding granulocyte-macrophage colony-stimulating factor [GM-CSF]) by the U.S. Food and Drug Administration (FDA) and European

Received 1 July 2021; accepted 29 September 2021;
https://doi.org/10.1016/j.omto.2021.09.008.

Correspondence: Toos Daemen, Department of Medical Microbiology and Infection Prevention, University Medical Center Groningen, University of Groningen, 9713 AV Groningen, the Netherlands.
E-mail: c.a.h.h.daemen@umcg.nl

This is an open access article under the CC BY license (http://creativecommons.org/licenses/by/4.0/).

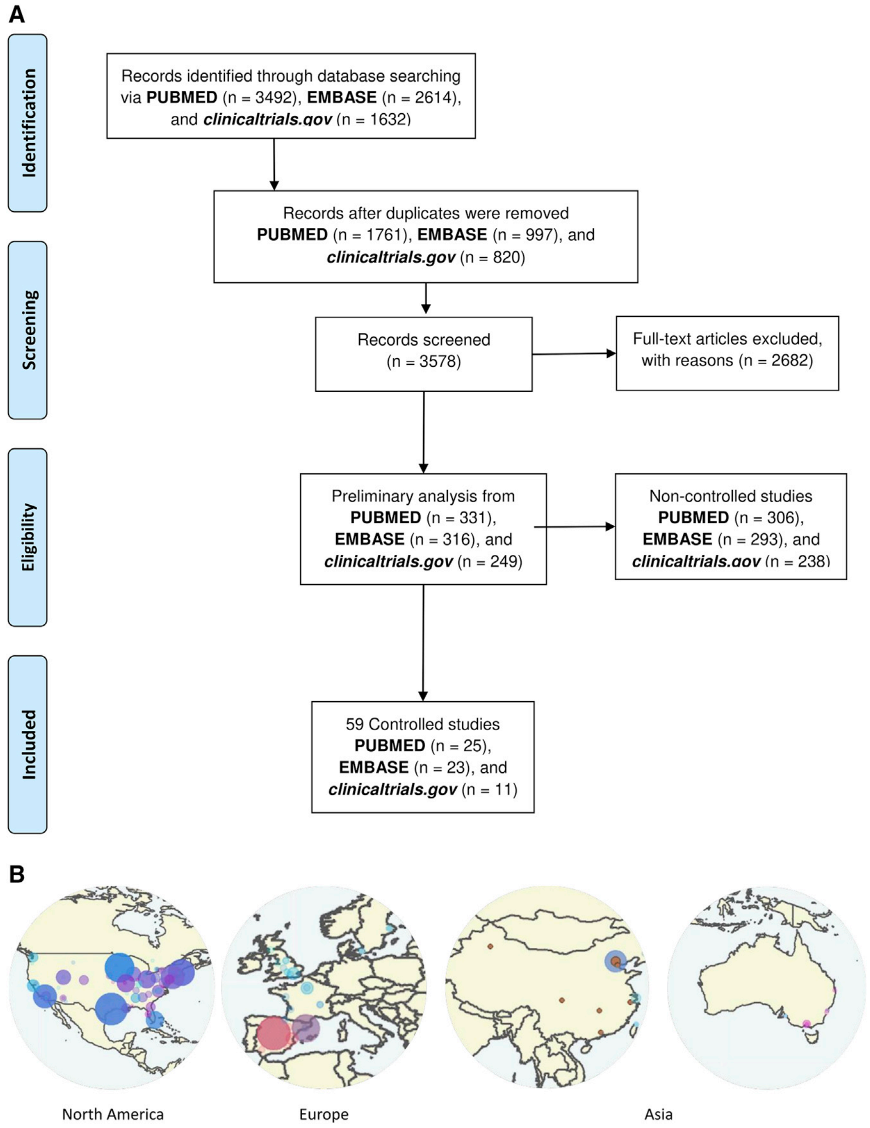

Figure 1. Screening of studies focusing on clinical safety and efficacy of onco-virotherapy

(A) Systematic review process and inclusion of articles and trials based on target criteria, where excluded reports were those that did not focus on the application of onco-virotherapy for cancer patients, or were they reviews, preclinical studies, or commentaries, or articles in which the abstract was not reported in English. (B) Geographical distribution of labs and institutes assessing safety and efficacy of onco-virotherapy.

Medicines Agency (EMA) in 2015, revived the interest for clinical applications of onco-virotherapy (Figure 2A). Most clinical studies have been conducted at phase I and II stages, often to test the safety and maximum tolerated dosage of the onco-virotherapy (Figure 2B). Although a wide range of viral vectors have been tested in phase I and II trials for safety,[5] few studies have progressed further to phase III trials (Figure 2B). In terms of the genetic nature, both enveloped and non-enveloped DNA and RNA vectors have been tested. Adenovirus (non-enveloped DNA virus) was the most commonly studied platform with 42.5% of studies, followed by herpes simplex virus (enveloped DNA virus) with 21.3% of studies, vaccinia virus (enveloped DNA virus) with 13.2% of studies, and reovirus (non-enveloped RNA virus) with 7.3% of studies. Patients with advanced and metastatic tumors were the most frequently recruited patients to receive onco-virotherapy (Figure 2C), likely due to the fact that cancer patients with a good prognosis generally benefit from standard care. However, this may limit our understanding of the potential efficacy of onco-virotherapy in patients with early stage cancers.

Although more than 200 trials related to onco-virotherapy are registered on ClinicalTrials.gov, fewer than 100 trials have been completed (Figure 2D). Many studies have reported being terminated or suspended due to funding issues or a lack of participants, and some trials are still active or recruiting patients. More than 2,000 cancer patients have been recruited and treated with onco-virotherapy, with phase I/II trials mostly conducted in a relatively small group of patients, and phase III trials with more than 200 patients per group (Figure 2E). Onco-virotherapy has been given to patients as a monotherapy, while occasionally it has been combined with chemotherapy and radiotherapy, and in some cases with targeted therapy (Figure 2F). Moreover, a limited number of case studies that did not have success with conventional checkpoint therapy or radiotherapy/chemotherapy (indicated as doctor's choice in Figure 2F) later proceeded with onco-virotherapy alone[11] or in combination with cyclophosphamide[12] to treat recurrent tumors in patients. Recent preclinical findings supporting the combination of immunotherapy have also led to clinical studies where checkpoint inhibitors targeting PD-1 (programmed death receptor-1) or PD-L1 (programmed death ligand-1) have been administered along with onco-virotherapy.[13]

Viral modifications and strategic therapy design to improve safety and efficacy

To establish the safety of onco-virotherapy for cancer patients, genetic modifications have been performed on a wide range of viral vectors to improve tumor targeting and attachment or to enhance tumor-specific replication (Figure 3A). Adenoviruses, herpes viruses, vaccinia viruses, and reoviruses have often been engineered to improve tumor specificity by such modifications. Improvement in targeting of adenovirus was for example achieved through knob modifications, and

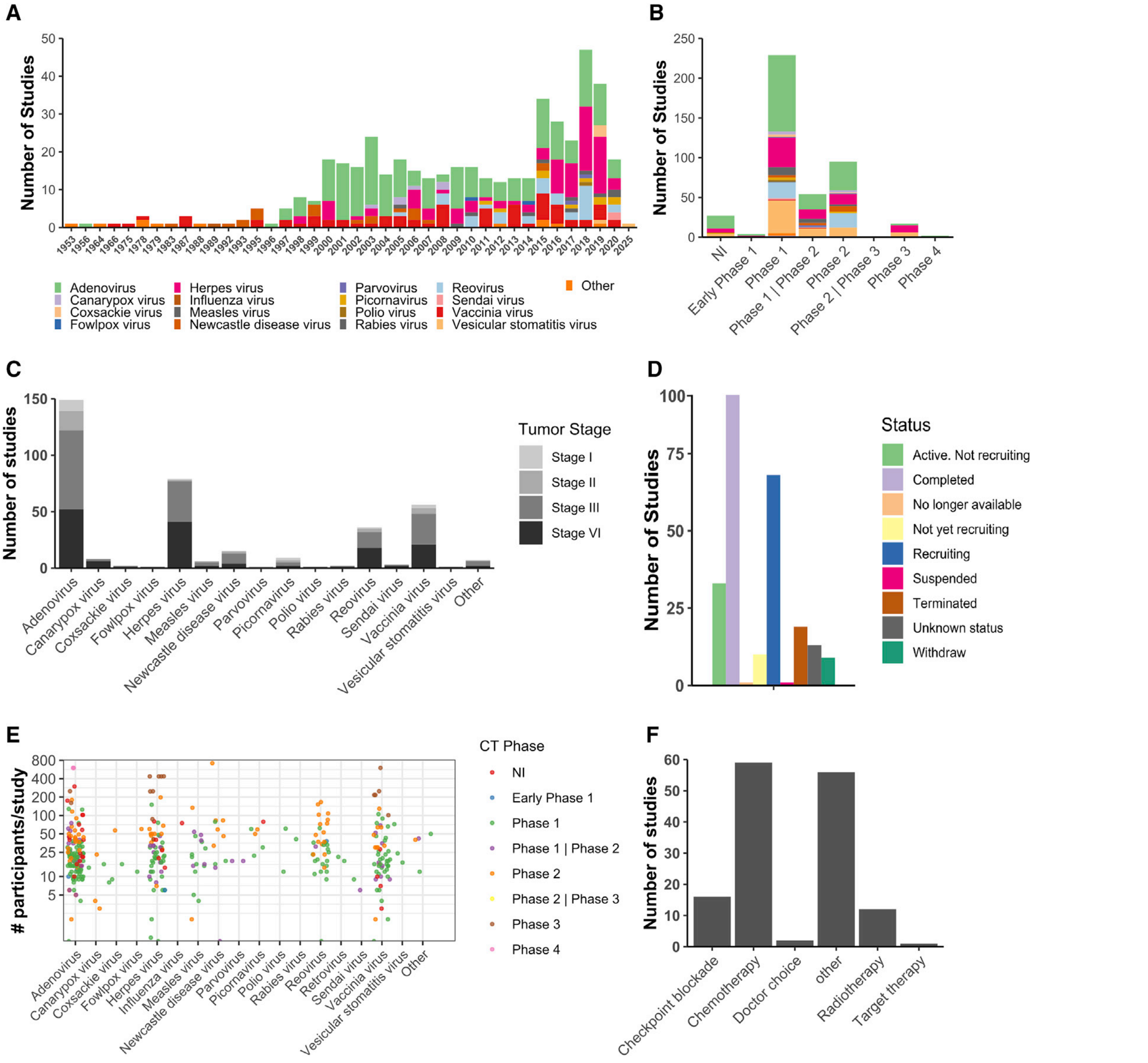

Figure 2. Scenario of clinical studies assessing onco-virotherapy

(A) Trends in clinical studies published as trials and articles assessing the role of different onco-virotherapy. (B) Frequency of studies published as trials and articles according to phase and type of onco-virotherapy studied, with the legend the same as in (A). (C) Patient tumor stage and status that received onco-virotherapy. (D) Onco-virotherapy trial status as per ClinicalTrials.gov. (E) Number of cancer patients recruited per study and treated via onco-virotherapy in different phases. (F) Frequency of therapeutic combination with onco-virotherapy.

attachment was improved by adenoviral fiber protein delta-24-RGD modification or via intercellular adhesion molecules. Alternatively, viral replication was restricted to tumor cells through modification or deletion of viral proteins such as early proteins (E1–E4) in adenovirus, and deletion of ICP34.5 and ICP47 in herpes virus (Table S1). Moreover, in some cases reovirus, vaccinia virus, and adenovirus were designed to have target-specific replication in tumor cells with differentially activated pathways such as RAS GTPases (rat sarcoma GTPases) or p16-RB (retinoblastoma protein) pathways (Table S1). The 2018 Nobel prize-winning technique of directed evolution has also been implemented as a means to screen adenoviruses with improved selectivity for tumor cells and their subsequent oncolysis.

Viral vectors have also been engineered to deliver and encode transgenes that act as "suicide-genetic switch" for controlled lysis of target cancer cells, for example by using ganciclovir to induce cell death of tumor cells expressing a thymidine kinase (TK2) transgene (Figure 3B). Simultaneously, viral vectors have been modified to improve efficacy by incorporating genes to enhance or direct antitumor immune responses. These are either tumor-specific antigens such as prostate-specific

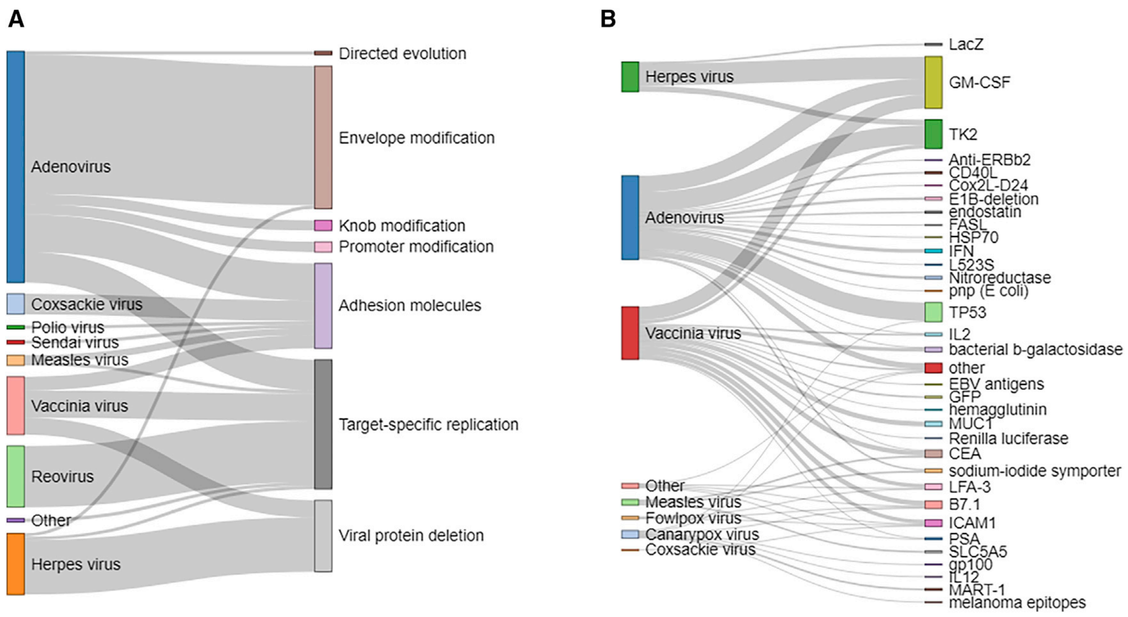

Figure 3. Viral modifications to improve safety and efficacy

(A) Virus modifications to improve tumor specificity. (B) Introduction of transgenes to improve therapeutic efficiency. Each line represents a single study (trial or article).

antigen (PSA) and mucin1 (MUC1), antitumor genes including tumor suppressor protein 53 (TP53), and genes encoding growth factors, cytokines, or ligand molecules, such as GM-CSF, interferon, interleukin-12, Fas ligand, and CD40 ligand, or marker genes encoding fluorescent proteins or enzymes that can be used for detection and quantification of transgene expression such as galactosidase and luciferase (Figure 3B). The most commonly encoded genes were found to be GM-CSF, TP53, and TK2, while adenoviruses, herpes viruses, and vaccinia viruses were the most frequently engineered vectors of choice.

Regarding therapeutic delivery, intratumoral delivery of viral vectors has remained the preferred route of injection due to safety and efficacy concerns by restricting viral infection to tumor. Nevertheless, intravenous, subcutaneous, and intramuscular routes have also been tested to achieve better biodistribution and to target distant metastatic sites (Figure 4A). In the case of melanoma, intratumoral delivery has remained a preferred choice due to easier accessibility of tumors. A wide range of virus doses have been tested for each viral vector type, and safety has been associated with lower doses, albeit at the cost of therapeutic efficacy. For example, adenoviruses have been given to patients at doses considered safe up to 10^{14} particles per injection for the best efficacy, whereas herpes viruses have demonstrated to be efficient in the range of 10^6–10^8 particles per injection (Figure 4B). In terms of the number of virus injections given to cancer patients, multiple injections were preferred and the scheme varied from daily, weekly, and monthly intervals (Figure 4C; Table S1).

Evaluation of clinical outcomes related to safety and efficacy

To test the therapeutic efficacy of viruses, studies have assessed different clinical outcomes such as overall survival, tumor size change, and overall response rate. However, as most of the trials comprised phase I/II stages, the most common study outcome was safety (Figure 5A). Although onco-virotherapy is nowadays also considered to induce antitumor immune responses, there have been relatively few (or have been fewer) studies assessing immunological outcomes (Figure 5A). Interestingly, the most commonly studied immunological features were antibody responses to viral vectors and antitumor adaptive responses mediated via lymphocytes (Figure 5B). Immune responses related to myeloid cells have been rarely assessed in clinical studies,[6,14–19] which might be due to difficulties in obtaining and processing clinical tissue samples from the patients as compared to the easier accessibility of peripheral blood to study lymphocytes, antibodies, and cytokine-based innate responses (Figure 5B).

Considering the controlled clinical trials, onco-virotherapy has often been compared with standard palliative care and/or treatment in addition to placebo groups of patients (Figure 5C). In the case of combinatorial therapeutic approaches, the control group was treated with virus alone. Onco-virotherapy, as compared to these standard treatments, either resulted in a better or similar outcome but rarely worsened the outcome as based on overall survival, progression-free survival, and decrease in tumor size (Figure 5D). The factors related to each of the controlled trials are summarized in Table 1. Of note, most trials did not involve control groups. However, also many of the trials with control groups had major limitations, as onco-virotherapy had to be compared to standard treatment or onco-virotherapy plus standard treatment. For example, in some cases the standard treatment was palliative care, placebo therapy, or observational data from tumor type-matched patients, which does not provide an indication of the improvement due to onco-virotherapy in comparison to conventional

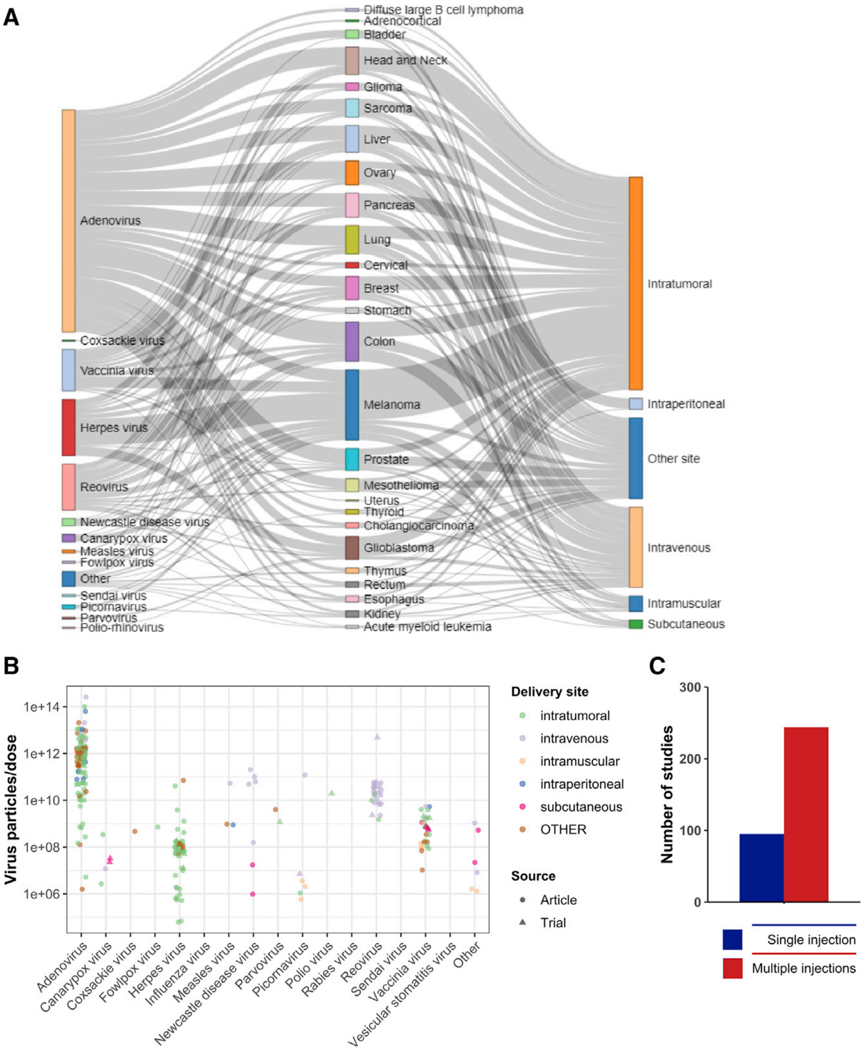

Figure 4. Strategic design to improve safety and efficacy

(A) Trends in choice of viral vector and delivery site according to tumor type. Each line represents a single study (trial or article). (B) Maximum tolerated dose per virus type in patients. (C) Frequency of injections applied.

chemotherapy and radiotherapy. Differences in individual trial design and the multitude of outcome measures make it difficult to compare studies performed by independent-unrelated institutes. Also, many articles and clinical trials have incomplete descriptions of the methods employed, increasing the difficulty of making comparisons and meta-analysis even more.

Published articles and clinical trials

Finally, as a reference to the design and conduct of future systematic analysis on clinical trials, we demonstrate the importance of including multiple databases to retrieve information. Through this study, we found that there were only a few variables showing similarity between the information collected via articles (obtained from PubMed and EMBASE) and trials (from ClinicalTrials.gov). Patient background-related information, such as age (Figure 6A), follow-up period (Figure 6B), and sex (Figure 6C), were equal between clinical trial and articles. However, other variables showed high disparity. Information related to trial design, such as the type of therapeutic combination, was found to be different between the data obtained from trials and articles (Figure 6D). Clinical trials were more often funded by private institutions, while articles more often received public funding (Figure 6E). Furthermore, the number of control groups was lower for the clinical trials compared to the articles (Figure 6F), and the number of study groups was smaller (Figure 6G). Clinical trials mainly focused on treating patients with advanced and metastatic cancer types, whereas articles also treated stage I and II cancer patients (Figure 6H) and investigated immune responses (Figures 6I and 6J). Although adenovirus was the most frequently studied viral vector by both clinical trials and articles, clinical trials studied herpes and vaccinia virus almost equally (Figure 6K). The described disparity is probably caused by the fact that many trials are not registered on ClinicalTrials.gov, and that articles on PubMed and EMBASE, in contrast to trials on ClinicalTrials.gov, are peer reviewed (Figure 1A).

DISCUSSION

Onco-virotherapy is a promising form of immunotherapy for the treatment of cancer. In this review we evaluated the clinical impact of onco-virotherapy for cancer patients who received virotherapy in comparison with cancer patients who received other therapies by means of a systematic analysis. Overall, our results indicate that onco-virotherapy has proven to be safe due to efforts in vector design, rational choices of therapeutic dosage, and delivery strategies. Simultaneously, various viral vectors have shown clinical efficacy in terms of better therapeutic outcomes as compared to standard care. Moreover, combinational strategies such as checkpoint blockade, chemotherapy, radiotherapy, and even introduction of immunogenic transgenes has improved clinical efficacy. With this analysis, we aim to provide a reference for clinicians and researchers in the onco-virotherapy field.

Our analysis identified 18 viral vectors that were used as therapeutic platform to treat 26 cancer types. These studies used the following virus types: adenovirus (42.5% of studies), canarypox virus (1.3% of studies), coxsackie virus (0.6% of studies), fowlpox virus (0.3% of studies), herpes virus (21.3% of studies), influenza virus (0.1% of

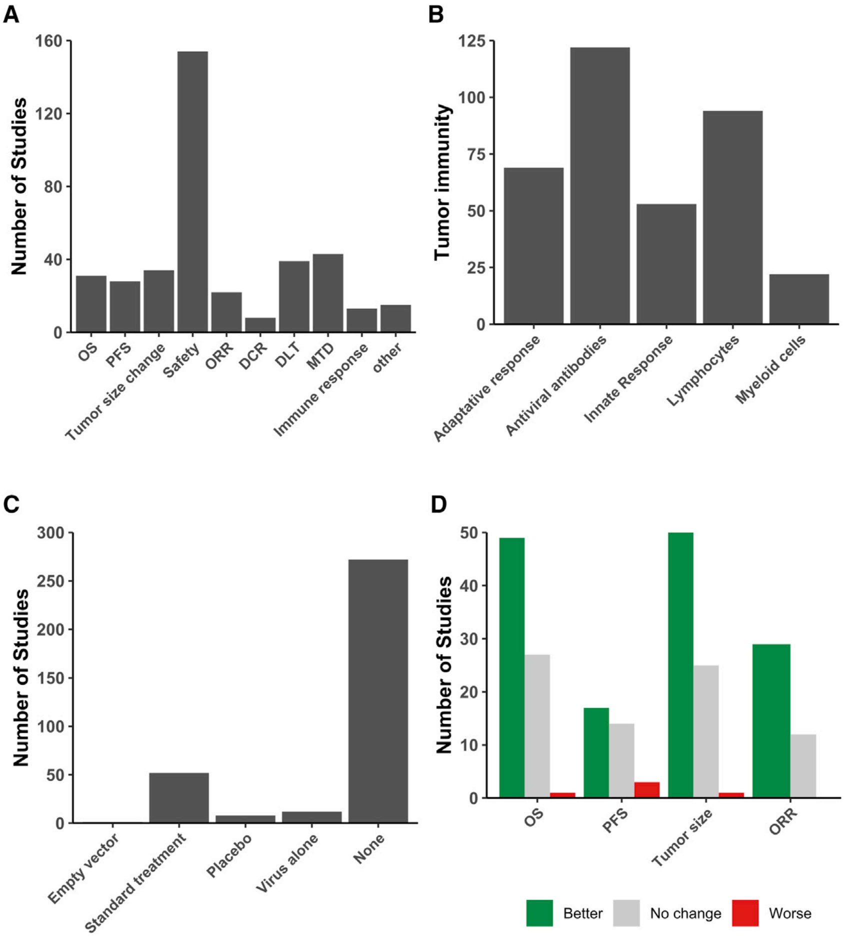

Figure 5. Clinical outcomes studied related to efficacy
(A) Frequent clinical outcomes studied after onco-virotherapy. (B) Type of immunological outcomes studied. (C) Commonly assessed control groups in comparison to onco-virotherapy. (D) Significant improvement or not in clinical outcomes after onco-virotherapy as compared to respective control groups. OS, overall survival; PFS, progression-free survival; ORR, objective response rate; DCR, disease control rate; DLT, dose limiting toxicity; MTD, maximum tolerated dose.

studies), measles virus (3% of studies), Newcastle disease virus (3.6% of studies), parvovirus (0.4% of studies), picornavirus (1.5% of studies), polio virus (0.4% of studies), rabies virus (0.1% of studies), retrovirus (0.3% of studies), reovirus (7.3% of studies), Semliki Forest virus (0.1% of studies), Sendai virus (0.3% of studies), vaccinia virus (13.2% of studies), and vesicular stomatitis virus (0.9% of studies). A wide range of cancers were treated in the clinics such as melanoma (17.1% of studies), colon cancer (9.7% of studies), lung cancer (7% of studies), head and neck cancer (6.8% of studies), liver cancer (6.5% of studies), ovarian cancer (5.9% of studies), pancreatic cancer (5.9% of studies), breast cancer (5.6% of studies), glioblastoma (5.6% of studies), prostate cancer (5.4% of studies), sarcoma (4.5% of studies), mesothelioma (3.2% of studies), bladder cancer (2% of studies), glioma (1.8% of studies), esophageal cancer (1.6% of studies), renal cancer (1.6% of studies), cervical cancer (1.4% of studies), cholangiocarcinoma (1.4% of studies), rectal cancer (1.4% of studies), stomach cancer (1.4% of studies), thymus cancer (1.4% of studies), thyroid cancer (1.1% of studies), diffuse large B cell lymphoma (0.7% of studies), acute myeloid leukemia (0.7% of studies), adrenocortical cancer (0.5% of studies), and uterine cancer (0.2% of studies). The clinical studies have been successful in recruiting patient irrespective of sex, age, and diversity in tumor types and stages. However, very few studies (<5% of studies) have evaluated the potential of onco-virotherapy on pediatric patients, whereas most (>80%) studies were focused on patients of 35–70 years in age.

Adenoviruses and herpesviruses were the most utilized virus types in clinical studies. Since 2015, the oncolytic herpes virus T-VEC is globally approved for the treatment of advanced melanoma.[62,63] However, the genetically modified adenovirus H101 (E1B-55K/E3B deletion), also known as Oncorine, was the very first oncolytic virus to be approved in 2005 in China for the treatment of head and neck cancer.[64,65] Moreover, adenoviruses have been extensively tested as gene therapy vectors, vaccine platforms, and synthetic biology tools, so for engineering, adenoviral vectors are more often chosen over other viruses as recently reviewed by Peter and Kühnel.[66] Similarly, viral vectors with an acceptable safety profile, such as the vaccinia virus and measles virus, have also been preferred choices for clinical testing.[1,67] Overall, this suggests that the development and easier accessibility of genetic engineering kits for vector modification has the potential to support the demand for novel viral therapeutics and their assessment in clinical research.

Strikingly, many viruses were not subjected to any genetic modification during the earlier years of onco-virotherapy development, although this approach has the potential to enhance the immunogenicity of viral vectors by the introduction of immunogenic genes. Nonetheless, onco-virotherapy modification gained popularity since 2000, and a large fraction of our analyzed trials were initiated in the years thereafter. Considering such native (non-modified) viruses, reovirus has been the most commonly used viral vector in onco-virotherapy that has not undergone genetic modifications.[68] Similarly, canarypox and fowlpox viruses have been used in their native form to deliver prostate tumor antigens, as they exhibit a weaker tropism to human cells and preferentially infect tumor cells.[69] Also, vesicular stomatitis virus is being exploited as therapeutic (ClinicalTrials.gov: NCT01628640 and NCT03120624) due to its sensitivity to

Table 1. Summary of controlled clinical trials exploring safety and efficacy of onco-virotherapy

Study	Virus type	Dose	Tumor type and stage	Mean age and sex	Control group	Transgene encoded	Tumor Specificity	Follow-up (months)	Endpoint	Outcome
1975, Everall et al.[20]	vaccinia virus	18	melanoma, stage 1	49.5 years, both	wide local excision	N/A	N/A	48	progression-free survival	no change
1989, Freedman et al.[21]	influenza A virus	N/A	uterine cervix carcinoma	46 years, female	radiotherapy	N/A	N/A	95	progression-free survival	no change
1992, Schlag et al.[22]	Newcastle disease virus	17	colorectal cancer with metastasis to the liver, stage 4	55 years, both	surgery	N/A	N/A	N/A	N/A	N/A
1993, Csatary et al.[23]	Newcastle disease virus	N/A	various cancers, stage 3	N/A, both	placebo	N/A	N/A	24	overall survival	better
1996, Ockert et al.[24]	Newcastle disease virus	N/A	colorectal carcinoma	N/A, both	surgery	N/A	N/A	22	safety	safe
1995, Hinkel et al.[25]	Newcastle disease virus	2^6	renal cell carcinoma, stage 3–4	N/A, both	uninfected irradiated renal carcinoma cells, virus alone, interleukin (IL)-2 alone	N/A	N/A	N/A	N/A	N/A
1995, Wallack et al.[26]	vaccinia virus	N/A	melanoma, stage 2	N/A, both	vaccinia virus alone	N/A	N/A	48	progression-free survival	no change
1997, Wallack et al.[27]	vaccinia virus	N/A	surgically resected melanoma, stage 2	N/A, both	vaccinia virus alone	N/A	N/A	42.28	overall survival	no change
1998, Wallack et al.[28]	vaccinia virus	N/A	melanoma, stage 3	N/A, both	vaccinia virus alone	N/A	N/A	46.3	N/A	no change
2000, Sandmair et al.[29]	adenovirus	310	glioma, stage 3–4	51 years, both	LacZ galactosidase	N/A	N/A	15	safety	safe
2000, Rainov et al.[3]	herpes virus	N/A	newly diagnosed, previously untreated glioblastoma multiforme	59.3 years, both	surgical resection and radiotherapy	thymidine kinase 2	deletion ICP34.5 and ICP47	N/A	Progression-free survival	no change
2002, Habib et al.[30]	adenovirus	311	hepatocellular carcinoma, stage 2	59 years, both	percutaneous ethanol injection	N/A	E1B deletion	1	safety	safe
2003, Voit et al.[31]	Newcastle disease virus	N/A	melanoma, stage 3	53.5 years, both	placebo	N/A	N/A	18	safety	safe
2003, Zhang et al.[32]	adenovirus	112	head and neck squamous cell carcinoma	N/A	radiotherapy	TP53	N/A	N/A	tumor size	better
2003, Chen C.[33]	adenovirus	N/A	nasopharyngeal carcinoma	N/A, both	radiotherapy	TP53	N/A	3	safety	safe
2004, Xia et al.[34]	adenovirus	1.512	squamous cell cancer of head and neck or esophagus	N/A, both	cisplatin with 5-fluorouracil or adriamycin with 5-fluorouracil	N/A	E1B-55-kDa gene deletion	N/A	objective response rate	better
2006, Spaner et al.[35]	canarypox	56	melanoma, stage 3–4	50 years, both	antigen peptides alone	gp100 antigen	N/A	8	N/A	N/A
2006, Lindsey et al.[36]	vaccinia virus	2^9	melanoma, stage 3–4	47 years, both	virus alone	tyrosinase	N/A	4	N/A	no change
2008, ClinicalTrials.gov: NCT00613509	canarypox	N/A	melanoma, stage 3–4	52.8 years, both	interferon alpha-2b	multiple melanoma antigens	N/A	88	progression-free survival	better

(*Continued on next page*)

Table 1. *Continued*

Study	Virus type	Dose	Tumor type and stage	Mean age and sex	Control group	Transgene encoded	Tumor Specificity	Follow-up (months)	Endpoint	Outcome
2008, Dong et al.[37]	adenovirus	2^{12}	lung, ovarian, liver, breast, celiothelioma, stage 3–4	59 years, both	cisplatin	TP53	N/A	2	objective response rate	better
2009, Guan et al.[38]	adenovirus	112	non-small cell lung cancer, stage 3–4	58 years, both	bronchial arterial infusion	TP53	N/A	12	safety	safe
2009, Pan et al.[39]	adenovirus	112	nasopharyngeal carcinoma, stage 2–4	48.5 years, both	radiotherapy	TP53	N/A	72	objective response rate	better
2009, Tian et al.[40]	adenovirus	112	hepatocellular carcinoma	55.5 years, both	transcatheter arterial chemoembolization	TP53	N/A	128	safety	safe
2010, Yang et al.[41]	adenovirus	312	hepatocellular carcinoma	55 years, both	fractionated stereotactic radiotherapy	TP53	N/A	35	safety	safe
2010, ClinicalTrials.gov: NCT01280058	reovirus	N/A	pancreatic cancer, stage 4	64 years, both	carboplatin with paclitaxel	N/A	RAS proto-oncogene dependency	48	progression-free survival	worse
2011, Heo et al.[42]	vaccinia virus	19	liver cancer	47.6 years, male	historical data of control patients or sorafenib alone	GM-CSF	EGFR-Ras dependency	2	tumor size	better
2011, Cerullo et al.[43]	adenovirus	112	advanced metastatic solid tumors, stage 3–4	61 years, both	cyclophosphamide in combination with virotherapy	GM-CSF	RGD-D24 targeting	12	overall survival	better
2012, Koski et al.[44]	adenovirus	111	colorectal, sarcoma, pancreatic, lung, breast, mesothelioma	57.5 years, both	verapamil	GM-CSF	integrin-targeted Ad5-D34-RGD	N/A	overall survival	no change
2013, Suriano et al.[45]	vaccinia virus	N/A	melanoma, stage 3	50 years, both	N/A	N/A	N/A	N/A	overall survival	no change
2013, Westphal et al.[46]	adenovirus	112	high-grade glioma, stage 3	55.4 years, both	resection and standard care	thymidine kinase 2	N/A	152	overall survival	no change
2014, Dong et al.[47]	adenovirus	112	unresectable hepatocellular carcinoma, stage 3-4	54 years, both	transarterial chemoembolization alone	N/A	N/A	N/A	progression-free survival	better
2014, Freytag et al.[48]	adenovirus	112	intermediate risk prostate cancer, stage 2	61 years, male	radiotherapy	thymidine kinase 2	N/A	48	safety	safe
2015 and 2018, ClincialTrials.gov: NCT00769704	herpes virus	48	melanoma, stage 3–4	64 years, both	GM-CSF therapy	GM-CSF	deletion of ICP34.5 and ICP47	44.4	other	N/A
2015, Donina et al.[49]	picornavirus	16	melanoma, stage 1–2	62.3 years, both	untreated observational group	N/A	N/A	47.8	overall survival	better
2015, ClincialTrials.gov: NCT00769703	herpes virus	18	melanoma, stage 3–4		GM-CSF therapy	GM-CSF	deletion of ICP34.5 and ICP47	44	disease control rate	better
2015, Kanerva et al.[50]	adenovirus	N/A	various cancers	60.5 years, both	matched controls, cancer type, and disease phase	GM-CSF	integrin-targeted	46	safety	safe
2015, Lin et al.[51]	adenovirus	112	hepatocellular carcinoma	55 years, both	transarterial chemoembolization of carboplatin	N/A	E1B deletion	12	overall survival	better

(*Continued on next page*)

Table 1. *Continued*

Study	Virus type	Dose	Tumor type and stage	Mean age and sex	Control group	Transgene encoded	Tumor Specificity	Follow-up (months)	Endpoint	Outcome
2015, ClinicalTrials.gov: NCT00179309	vaccinia virus	19	breast cancer, stage 4	54.3 years, both	docetaxel	CEA, MUC1, and TRICOM	CEA and MUC1	197	progression-free survival	better
2015, ClinicalTrials.gov: NCT00634595	adenovirus	112	head and neck squamous cell carcinoma, stage 3–4	52 years, both	cisplatin and paclitaxel	endostatin	N/A	10	N/A	no change
2016, ClinicalTrials.gov: NCT02705196	adenovirus	N/A	pancreatic cancer, stage 3	>18 years, both	gemcitabine and paclitaxel with and without anti-PD-L1 antibody	N/A	N/A	N/A	N/A	N/A
2016, Andtbacka et al.[10,52]	herpes virus	N/A	melanoma, stage 3–4	63 years, both	subcutaneous injection of GM-CSF	GM-CSF	deletion of ICP34.5 and ICP47	N/A	tumor size	better
2016, Gao et al.[53]	adenovirus	510	malignant and solid tumors, stage 3–4	35 years, both	adriamycin alone	GM-CSF	N/A	N/A	N/A	better
2016, ClinicalTrials.gov: NCT00870181	adenovirus	112	high-grade gliomas, stage 3–4	52.5 years, both	surgery, systemic chemotherapy, or palliative care	thymidine kinase 2	N/A	71	progression-free survival	better
2016, Andtbacka et al.[10,52]	herpes virus	1^{8}	unresected melanoma, stage 3–4	63 years, both	GM-CSF therapy	GM-CSF	deletion of ICP34.5 and ICP47	30	disease control rate	N/A
2016, et al.[54]	adenovirus	1^{12}	cervical cancer, stage 2–3	52 years, female	radiotherapy in combination with brachytherapy	TP53	N/A	605	safety	safe
2016, Harrington et al.[55]	herpes virus	1^{8}	melanoma, stage 3–4	63 years, both	GM-CSF therapy	GM-CSF	deletion of ICP34.5 and ICP47	18	overall survival	better
2017, Ma et al.[56]	adenovirus	1^{12}	nasopharyngeal carcinoma, stage 2	N/A, both	radiation, cisplatin or 5-fluorouracil	TP53	N/A	36	overall survival	better
2017, Cohn et al.[57]	reovirus	3^{10}	ovarian, tubal, or peritoneal cancer, stage 2–3	60 years, female	paclitaxel	N/A	N/A	128	overall survival	no change
2018, Bradbury et al.[58]	reovirus	4.5^{12}	lung adenocarcinoma, stage 3–4	64 years, both	chemotherapy	N/A	N/A	180	overall survival	no change
2018, Xiao et al.[4]	adenovirus	112	advanced unresectable soft-tissue sarcomas, stage 3	49 years, both	hyperthermia alone or in combination with radiotherapy	TP53	N/A	N/A	disease control rate	better
2018, Liu et al.[59]	adenovirus	19	hypopharyngeal squamous cell carcinoma	57.9 years, both	surgery alone or in combination with chemo-radiotherapy	TP53	N/A	36	overall survival	better
2018, ClinicalTrials.gov: NCT01622543	reovirus	310	colorectal cancer, stage 4	50 years, both	leucovorin, 5-fluorouracil, oxaliplatin, or bevacizumab	N/A	N/A	13	progression free survival	worse
2018, ClinicalTrials.gov: NCT01619813	reovirus	310	metastatic prostate adeno-carcinoma, stage 4	69 years, male	docetaxel and prednisone	N/A	N/A	20	progression-free survival	better
2018, ClinicalTrials.gov: NCT01656538	reovirus	310	metastatic breast cancer, stage 4	44 years, female	paclitaxel	N/A	N/A	295	progression-free survival	no change
2018, ClinicalTrials.gov: NCT01708993	reovirus	4.510	on-small cell lung cancer, stage 3-4	63 years, both	pemetrexed or docetaxel	N/A	N/A	27	safety	safe
2018, ClinicalTrials.gov: NCT01740297	herpes virus	48	unresectable melanoma, stage 3-4	64.5 years, both	ipilimumab	GM-CSF	deletion of ICP34.5 and ICP47	156	safety	safe

(*Continued on next page*)

Table 1. Continued

Study	Virus type	Dose	Tumor type and stage	Mean age and sex	Control group	Transgene encoded	Tumor Specificity	Follow-up (months)	Endpoint	Outcome
2018, He et al.[60]	adenovirus	112	hepatocellular carcinoma, stage 1–4	55 years, both	transarterial chemo-embolization	N/A	N/A	13	overall survival	better
2019, NCT01387555	vaccinia virus	19	hepatocellular carcinoma, stage 3	57 years, both	supportive care	GM-CSF	N/A	4.3	overall survival	no change
2020, Schenk et al.[61]	picornavirus	111	small cell carcinoma	63 years, both	saline	N/A	natural	17	progression-free survival	worse

N/A, not available; GM-CSF, granulocyte-macrophage colony-stimulating factor; TP53, tumor protein 53; ICP, infected cell protein.

interferon-mediated antiviral responses exhibited by normal cells, which allows preferential infection and lysis of tumor cells devoid of active interferon responses.[70]

Many of the utilized viral vectors were administered in combination with another form of therapy such as chemotherapy,[34,40,42,43,57,71], immune checkpoint inhibitors,[13,72,73] or radiotherapy.[3,4,32,59] Furthermore, most viruses were injected intratumorally and were administered multiple times, which is not surprising, as intratumoral administration of viruses has been shown to be effective. Additionally, multiple injections increase the possibility of inducing stronger anti-tumor effects and related immune responses.[74] However, multiple intratumoral injections also face the limitations of an invasive approach, for example in patients with glioblastoma,[75] or in pediatric patients,[76,77] thus indicating room for improvement.[78] Furthermore, immune responses such as virus neutralization by antiviral antibodies,[79] neutralization mediated by complement activation,[80,81] and cellular-antiviral responses mediated by natural killer (NK) cells[82] and T cells also prove to be a challenge to onco-virotherapy. This has led to an increased requirement of dosage during subsequent rounds of treatment to counterbalance virus elimination in patients.

In addition to improving the safety and efficacy of viral vectors via genetic modifications and combinatorial approaches, efforts have also been made in overcoming the challenges related to manufacturing a clinical-grade stock of these viruses. Factors such as ensuring sterility and proper handling during production, improvement of virus yields, appropriate purification strategies, and formulation for long-term stability and storage have been discussed in detail in existing literature.[83,84] Moreover, regulatory aspects ranging from virus design and production up to therapeutic utilization in clinics have remained of immense importance for safe application of onco-virotherapy.[85] Taken together, these factors can potentially influence the feasibility of producing the maximum dose required for patient treatment, especially in the case of multiple injections and for virus types that require high dose for effective therapy. Manufacturing challenges can also impact the cost of therapy where the economic evaluation of onco-virotherapy has yet to demonstrate itself as a less expensive alternative to existing therapies.[86,87]

Non-randomized cohort studies and non-controlled trials have primarily focused on assessing the safety profile of the viral vector, and therefore determinations of dose limiting toxicity and maximum tolerated dose have remained important. Additionally, side effects such as fever, fatigue, flu-like symptoms, nausea, and pain at the site of injection, among others, have also been reported to occur after onco-virotherapy, although rarely in severe form.[6]

In most controlled trials, onco-virotherapy treatment resulted in better outcomes for individual variables (>70% of controlled trials) or no change (>40% of controlled trials), although some trials reported worse outcomes (Table 1). This indicates that further improvements in onco-virotherapy are still needed. We found that most of the studies did not include the immune response in their outcome

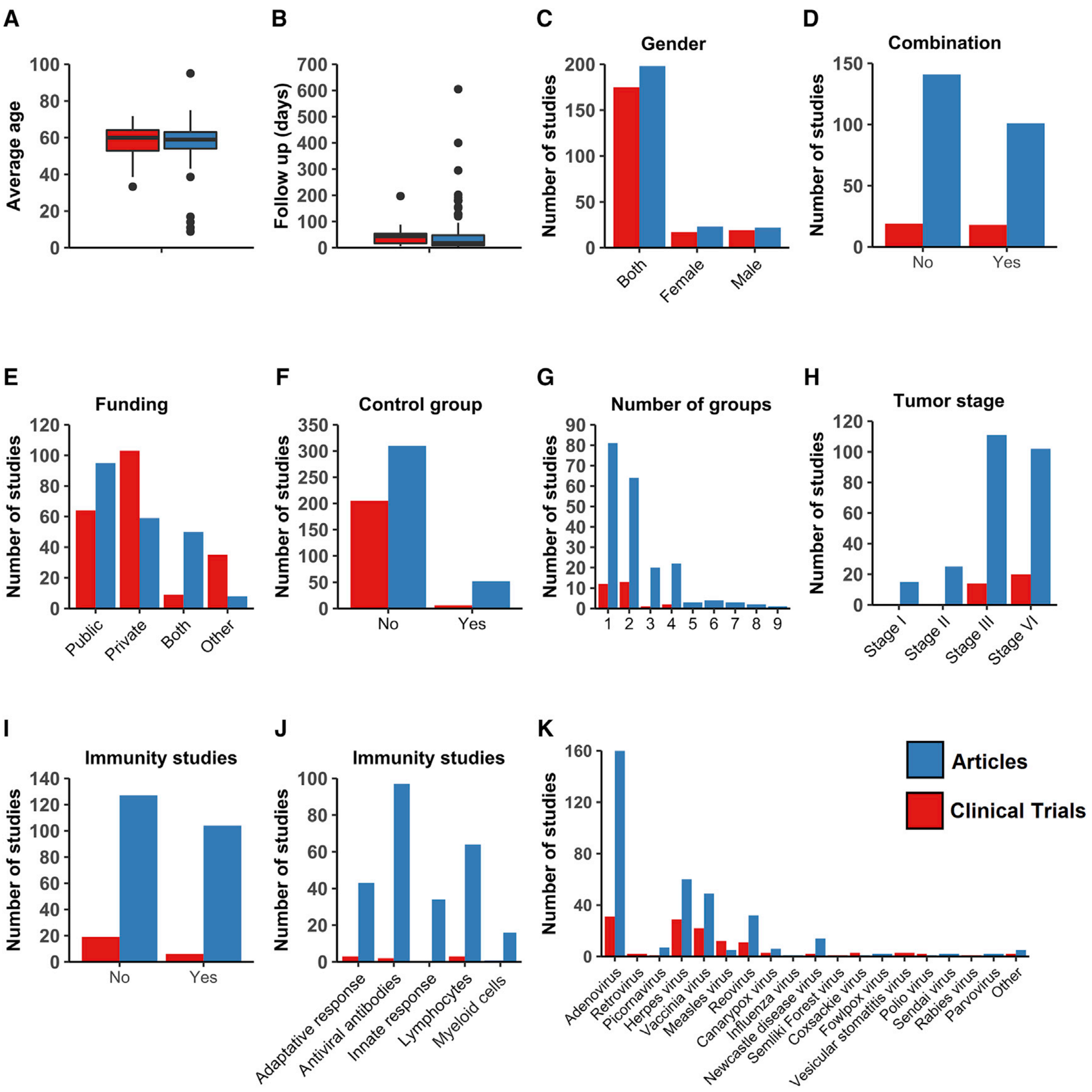

Figure 6. Disparity of data obtained from clinicaltrials.gov and articles

Disparities are reported in terms of (A) patient age, (B) follow-up days, (C) sex, (D) combinatorial strategy with onco-virotherapy, (E) funding, (F) inclusion of control group, (G) number of groups per study, (H) patient tumor stage, (I) immunological outcomes studied, (J) type of immunological outcomes studied, and (K) type of onco-virotherapy studied. Bars in red represent clinical trials and in blue represent articles (retrieved from PubMed and EMBASE).

measures, which was unexpected and remarkable, as immunogenic effects are characteristic for onco-virotherapy. Instead, most trials focused on general outcome measures such as progression-free survival, overall survival, and tumor size change. Hence, it would be important that in the future onco-virotherapy clinical trials also assess the immune response as an outcome measure. Moreover, most of the trials have chosen clinical criteria of assessment based on the published guidelines such as RECIST.[6] However, recent literature[88] and our review indicate the need to establish new parameters to evaluate tumor response to virotherapy in terms of immune response, reduction of metastasis, and alteration in tumor metabolism and growth.

Only a limited number of trials compared the efficacy of onco-virotherapy with conventional treatments such as chemotherapy or radiotherapy (Table 1). For example, the FDA approved therapeutic T-VEC, which is administered intratumorally, has only been studied in comparison with intravenous GM-CSF injections, where T-VEC showed significantly better outcomes.[63] So far the performance of T-VEC in comparison with chemotherapy and/or radiotherapy has not been investigated. Interestingly, recent trials in which T-VEC was combined with checkpoint immunotherapy resulted in better outcomes as compared to monotherapy of either T-VEC or checkpoint immunotherapy.[13] Again, this emphasizes the importance of assessing immune responses after onco-virotherapy.

From the more than 20 different solid tumor types evaluated, skin cutaneous melanoma was most commonly studied. This can likely be ascribed to the fact that this tumor type is accessible for intratumoral injection without the need of surgical interventions. Also, most melanoma cells contain a high mutational burden,[89] and this increases the likelihood of tumor-specific antigen release into the tumor microenvironment upon oncolysis, thereby improving the potency of the onco-virotherapy. Additionally, most tumors studied were in an advanced or metastatic state, likely due to the fact that cancer patients with a good prognosis generally benefit from standard care. This is probably a confounding factor during the assessment of onco-virotherapy, as advanced patients are generally less likely to respond to any therapeutic intervention. Therefore, including patients who suffer from early and localized cancer, as well as high-risk or early refractory patients, may be a strategy to further explore the effectiveness of onco-virotherapy, as these patients would be more likely to benefit.

Finally, we aspire that the information gathered here can be used as a starting point to construct an interactive database that provides information to clinicians and researchers who are interested in the therapeutic potential of onco-virotherapy. Moreover, our search strategies can be used to regularly update such a database by collecting and screening trial-related data from ClinicalTrials.gov and articles from PubMed and EMBASE. Furthermore, we encourage clinicians and researchers to continue reviewing literature associated with clinical research by assessing multiple platforms, as it increases the possibilities of finding trial results and articles that are exclusive to a particular platform.

MATERIALS AND METHODS

Protocol and eligibility

We used the preferred reporting items for systematic review and meta-analysis protocol (PRISMA-P) statement as a guide for our analysis. To define our research question, we utilized the PICOS (patient, intervention, comparator, outcome, study type) framework based on the accepted PRISMA guidelines. Accordingly, we focused on cancer patients (P) who receive onco-virotherapy (I) compared with patients who receive other therapies (including placebo, chemotherapy, immunotherapy, radiotherapy) (C), the clinical impact with respect to response rate (O1) or tumor size change (O2) or safety (O3), and such (O4-to-n), and in a clinical setup (S) for therapeutic purposes.

Search strategy and screening of articles

We retrieved clinical trials from the ClinicalTrials.gov registry (https://clinicaltrials.gov) and PubMed (https://PubMed.ncbi.nlm.nih.gov) and EMBASE (https://www.embase.com) databases until August 2020. For each medium, we used a different search strategy, as specified in the Supplemental information. Through the search strategy, we found 3,492 articles from PubMed, 2,614 articles from EMBASE, and 1,632 trials from ClinicalTrials.gov. After the removal of duplicates, at least two authors manually screened the retrieved articles for inclusion, where we excluded articles or trials that did not focus on the application of onco-virotherapy for cancer patients, articles that were reviews, preclinical studies or commentaries, and articles in which the abstract was not reported in English (Figure 1). Any conflicts were resolved through discussion. This allowed us to include 331 articles from PubMed, 316 articles from EMBASE, and 249 trials from ClinicalTrials.gov. Subsequently, we added all articles in our database using browser-based REDCap software (Vanderbilt University, Nashville, TN, USA). All of the data and results are provided in Table S1 and are intended to serve as a resource for future studies.

Preliminary qualitative analysis and screening of controlled clinical studies

To observe the trends in clinical studies exploring the potential of onco-virotherapy for cancer treatment, we did a preliminary analysis of studies including retrieved data from articles and trials (Figures 1, 2, 3, 4, and 5). This preliminary analysis was based on the literature found via PubMed (331 articles), EMBASE (316 articles), and ClinicalTrials.gov (249 trials) as described earlier. Finally, we identified and summarized 59 controlled clinical studies reporting comparative data from respective articles and trials (Table 1). All figures and tables were made using ggplot2, or networkD3 R packages,[90] and the graphical abstract was made using BioRender.

SUPPLEMENTAL INFORMATION

Supplemental information can be found online at https://doi.org/10.1016/j.omto.2021.09.008.

ACKNOWLEDGMENTS

This work was supported by CNPq (grant award 305700/2017-0 to R.C.), CAPES (finance code 001, to D.K.B.), and by an ATTP-GSMS scholarship (Abel Tasman Talent Program to D.K.B.). The authors would like to thank Dr. Peter G. Braun and Sjoukje van der Werf from the university library at the University of Groningen for support in formulation of the search strategy for the systematic review. Moreover, the authors thank the reviewers for their valuable feedback regarding the manuscript.

AUTHOR CONTRIBUTIONS

All authors made substantial contributions to the manuscript. Conception and design, D.K.B., L.W., and T.D.; supervision and funding acquisition, T.D. and R.C.; collection and assembly of data, D.K.B., L.W., and L.R.C.B.; data analysis and interpretation, D.K.B., L.W., L.R.C.B., T.D., and R.C.; manuscript writing, D.K.B., L.W., L.R.C.B., R.C., and T.D. All authors reviewed the manuscript and approved the final version for submission.

DECLARATION OF INTERESTS

The authors declare no competing interests.

REFERENCES

1. Guo, Z.S., Lu, B., Guo, Z., Giehl, E., Feist, M., Dai, E., Liu, W., Storkus, W.J., He, Y., Liu, Z., and Bartlett, D.L. (2019). Vaccinia virus-mediated cancer immunotherapy: cancer vaccines and oncolytics. J. Immunother. Cancer *7*, 6.

2. Wold, W.S.M., and Toth, K. (2013). Adenovirus vectors for gene therapy, vaccination and cancer gene therapy. Curr. Gene Ther. *13*, 421–433.

3. Rainov, N.G. (2000). A phase III clinical evaluation of herpes simplex virus type 1 thymidine kinase and ganciclovir gene therapy as an adjuvant to surgical resection and radiation in adults with previously untreated glioblastoma multiforme. Hum. Gene Ther. *11*, 2389–2401.

4. Xiao, S.W., Xu, Y.-Z., Xiao, B.-F., Jiang, J., Liu, C.-Q., Fang, Z.W., Li, D.-M., Li, X.F., Cai, Y., Li, Y.H., et al. (2018). Recombinant adenovirus-p53 gene therapy for advanced unresectable soft-tissue sarcomas. Hum. Gene Ther. *29*, 699–707.

5. Buijs, P.R.A., Verhagen, J.H.E., van Eijck, C.H.J., and van den Hoogen, B.G. (2015). Oncolytic viruses: From bench to bedside with a focus on safety. Hum. Vaccin. Immunother. *11*, 1573–1584.

6. Macedo, N., Miller, D.M., Haq, R., and Kaufman, H.L. (2020). Clinical landscape of oncolytic virus research in 2020. J. Immunother. Cancer *8*, e001486.

7. Martin, N.T., and Bell, J.C. (2018). Oncolytic virus combination therapy: Killing one bird with two stones. Mol. Ther. *26*, 1414–1422.

8. Cook, M., and Chauhan, A. (2020). Clinical application of oncolytic viruses: A systematic review. Int. J. Mol. Sci. *21*, 7505.

9. Li, Z., Jiang, Z., Zhang, Y., Huang, X., and Liu, Q. (2020). Efficacy and safety of oncolytic viruses in randomized controlled trials: A systematic review and meta-analysis. Cancers (Basel) *12*, 1416.

10. Andtbacka, R.H.I., Agarwala, S.S., Ollila, D.W., Hallmeyer, S., Milhem, M., Amatruda, T., Nemunaitis, J.J., Harrington, K.J., Chen, L., Shilkrut, M., et al. (2016). Cutaneous head and neck melanoma in OPTiM, a randomized phase 3 trial of talimogene laherparepvec versus granulocyte-macrophage colony-stimulating factor for the treatment of unresected stage IIIB/IIIC/IV melanoma. Head Neck *38*, 1752–1758.

11. Westbrook, B.C., Norwood, T.G., Terry, N.L.J., McKee, S.B., and Conry, R.M. (2019). Talimogene laherparepvec induces durable response of regionally advanced Merkel cell carcinoma in 4 consecutive patients. JAAD Case Rep. *5*, 782–786.

12. Vassilev, L., Ranki, T., Joensuu, T., Jäger, E., Karbach, J., Wahle, C., Partanen, K., Kairemo, K., Alanko, T., Turkki, R., et al. (2015). Repeated intratumoral administration of ONCOS-102 leads to systemic antitumor $CD8^+$ T-cell response and robust cellular and transcriptional immune activation at tumor site in a patient with ovarian cancer. OncoImmunology *4*, e1017702.

13. Chesney, J., Puzanov, I., Collichio, F., Singh, P., Milhem, M.M., Glaspy, J., Hamid, O., Ross, M., Friedlander, P., Garbe, C., et al. (2018). Randomized, open-label phase ii study evaluating the efficacy and safety of talimogene laherparepvec in combination with ipilimumab versus ipilimumab alone in patients with advanced, unresectable melanoma. J. Clin. Oncol. *36*, 1658–1667.

14. Taipale, K., Liikanen, I., Koski, A., Heiskanen, R., Kanerva, A., Hemminki, O., Oksanen, M., Grönberg-Vähä-Koskela, S., Hemminki, K., Joensuu, T., and Hemminki, A. (2016). Predictive and prognostic clinical variables in cancer patients treated with adenoviral oncolytic immunotherapy. Mol. Ther. *24*, 1323–1332.

15. Everett, A.S., Pavlidakey, P.G., Contreras, C.M., De Los Santos, J.F., Kim, J.Y., McKee, S.B., Kaufman, H.L., and Conry, R.M. (2018). Chronic granulomatous dermatitis induced by talimogene laherparepvec therapy of melanoma metastases. J. Cutan. Pathol. *45*, 48–53.

16. Hemminki, O., Oksanen, M., Taipale, K., Liikanen, I., Koski, A., Joensuu, T., Kanerva, A., and Hemminki, A. (2018). Oncograms visualize factors influencing long-term survival of cancer patients treated with adenoviral oncolytic immunotherapy. Mol. Ther. Oncolytics *9*, 41–50.

17. Hu, J.C.C., Coffin, R.S., Davis, C.J., Graham, N.J., Groves, N., Guest, P.J., Harrington, K.J., James, N.D., Love, C.A., McNeish, I., et al. (2006). A phase I study of $\text{OncoVEX}^{\text{GM-CSF}}$, a second-generation oncolytic herpes simplex virus expressing granulocyte macrophage colony-stimulating factor. Clin. Cancer Res. *12*, 6737–6747.

18. Kaufman, H.L., Kim, D.W., DeRaffele, G., Mitcham, J., Coffin, R.S., and Kim-Schulze, S. (2010). Local and distant immunity induced by intralesional vaccination with an oncolytic herpes virus encoding GM-CSF in patients with stage IIIc and IV melanoma. Ann. Surg. Oncol. *17*, 718–730.

19. Hwang, T.-H., Moon, A., Burke, J., Ribas, A., Stephenson, J., Breitbach, C.J., Daneshmand, M., De Silva, N., Parato, K., Diallo, J.-S., et al. (2011). A mechanistic proof-of-concept clinical trial with JX-594, a targeted multi-mechanistic oncolytic poxvirus, in patients with metastatic melanoma. Mol. Ther. *19*, 1913–1922.

20. Everall, J.D., O'Doherty, C.J., Wand, J., and Dowd, P.M. (1975). Treatment of primary melanoma by intralesional vaccinia before excision. Lancet *2*, 583–586.

21. Freedman, R.S., Bowen, J.M., Atkinson, E.N., Wallace, S., Lotzová, E., Silva, E., Edwards, C.L., Delclos, L., Scott, W., Patenia, B., et al. (1989). Randomized comparison of viral oncolysate plus radiation and radiation alone in uterine cervix carcinoma. Am. J. Clin. Oncol. *12*, 244–250.

22. Schlag, P., Manasterski, M., Gerneth, T., Hohenberger, P., Dueck, M., Herfarth, C., Liebrich, W., and Schirrmacher, V. (1992). Active specific immunotherapy with Newcastle-disease-virus-modified autologous tumor cells following resection of liver metastases in colorectal cancer. First evaluation of clinical response of a phase II-trial. Cancer Immunol. Immunother. *35*, 325–330.

23. Csatary, L.K., Eckhardt, S., Bukosza, I., Czegledi, F., Fenyvesi, C., Gergely, P., Bodey, B., and Csatary, C.M. (1993). Attenuated veterinary virus vaccine for the treatment of cancer. Cancer Detect. Prev. *17*, 619–627.

24. Ockert, D., Schirrmacher, V., Beck, N., Stoelben, E., Ahlert, T., Flechtenmacher, J., Hagmüller, E., Buchcik, R., Nagel, M., and Saeger, H.D. (1996). Newcastle disease virus-infected intact autologous tumor cell vaccine for adjuvant active specific immunotherapy of resected colorectal carcinoma. Clin. Cancer Res. *2*, 21–28.

25. Hinkel, A., de Riese, W., Alefelder, J., Laumann, D., Falkenberg, F.W., Senge, T., and Hertle, L. (1995). Active specific immunotherapy with an autologous virus-modified tumour cell vaccine in human renal cell carcinoma (RCC). Eur. J. Cancer *31A*, 1719–1720.

26. Wallack, M.K., Sivanandham, M., Balch, C.M., Urist, M.M., Bland, K.I., Murray, D., Robinson, W.A., Flaherty, L.E., Richards, J.M., Bartolucci, A.A., et al. (1995). A phase III randomized, double-blind multiinstitutional trial of vaccinia melanoma oncolysate-active specific immunotherapy for patients with stage II melanoma. Cancer *75*, 34–42.

27. Wallack, M.K., Sivanandham, M., Ditaranto, K., Shaw, P., Balch, C.M., Urist, M.M., Bland, K.I., Murray, D., Robinson, W.A., Flaherty, L., et al. (1997). Increased survival of patients treated with a vaccinia melanoma oncolysate vaccine: Second interim analysis of data from a phase III, multi-institutional trial. Ann. Surg. *226*, 198–206.

28. Wallack, M.K., Sivanandham, M., Balch, C.M., Urist, M.M., Bland, K.I., Murray, D., Robinson, W.A., Flaherty, L., Richards, J.M., Bartolucci, A.A., and Rosen, L. (1998). Surgical adjuvant active specific immunotherapy for patients with stage III melanoma: The final analysis of data from a phase III, randomized, double-blind, multicenter vaccinia melanoma oncolysate trial. J. Am. Coll. Surg. *187*, 69–77, discussion 77–79.

29. Sandmair, A.-M., Loimas, S., Puranen, P., Immonen, A., Kossila, M., Puranen, M., Hurskainen, H., Tyynelä, K., Turunen, M., Vanninen, R., et al. (2000). Thymidine kinase gene therapy for human malignant glioma, using replication-deficient retroviruses or adenoviruses. Hum. Gene Ther. *11*, 2197–2205.

30. Habib, N., Salama, H., Abd El Latif Abu Median, A., Isac Anis, I., Abd Al Aziz, R.A., Sarraf, C., Mitry, R., Havlik, R., Seth, P., Hartwigsen, J., et al. (2002). Clinical trial of E1B-deleted adenovirus (dl1520) gene therapy for hepatocellular carcinoma. Cancer Gene Ther. *9*, 254–259.

31. Voit, C., Kron, M., Schwurzer-Voit, M., and Sterry, W. (2003). Intradermal injection of Newcastle disease virus-modified autologous melanoma cell lysate and interleukin-2 for adjuvant treatment of melanoma patients with resectable stage III disease. J. Dtsch Dermatol. Ges. *1*, 120–125.

32. Zhang, S.W., Xiao, S.W., Liu, C.Q., Sun, Y., Su, X., Li, D.M., Xu, G., Cai, Y., Zhu, G.Y., Xu, B., and Lü, Y.Y. (2003). [Treatment of head and neck squamous cell carcinoma by recombinant adenovirus-p53 combined with radiotherapy: A phase II clinical trial of 42 cases]. Zhonghua Yi Xue Za Zhi *83*, 2023–2028.

33. Chen, C.B., Pan, J.J., and Xu, L.Y. (2003). [Recombinant adenovirus p53 agent injection combined with radiotherapy in treatment of nasopharyngeal carcinoma: a phase II clinical trial]. Zhonghua Yi Xue Za Zhi *83*, 2033–2035.

34. Xia, Z.-J., Chang, J.-H., Zhang, L., Jiang, W.-Q., Guan, Z.-Z., Liu, J.-W., Zhang, Y., Hu, X.-H., Wu, G.-H., Wang, H.-Q., et al. (2004). [Phase III randomized clinical trial of intratumoral injection of E1B gene-deleted adenovirus (H101) combined with cisplatin-based chemotherapy in treating squamous cell cancer of head and neck or esophagus]. Ai Zheng*23*, pp. 1666–1670.

35. Spaner, D.E., Astsaturov, I., Vogel, T., Petrella, T., Elias, I., Burdett-Radoux, S., Verma, S., Iscoe, N., Hamilton, P., and Berinstein, N.L. (2006). Enhanced viral and tumor immunity with intranodal injection of canary pox viruses expressing the melanoma antigen, gp100. Cancer *106*, 890–899.

36. Lindsey, K.R., Gritz, L., Sherry, R., Abati, A., Fetsch, P.A., Goldfeder, L.C., Gonzales, M.I., Zinnack, K.A., Rogers-Freezer, L., Haworth, L., et al. (2006). Evaluation of prime/boost regimens using recombinant poxvirus/tyrosinase vaccines for the treatment of patients with metastatic melanoma. Clin. Cancer Res. *12*, 2526–2537.

37. Dong, M., Li, X., Hong, L.-J., Xie, R., Zhao, H.-L., Li, K., Wang, H.-H., Shin, W.-D., and Shen, H.-J. (2008). Advanced malignant pleural or peritoneal effusion in patients treated with recombinant adenovirus p53 injection plus cisplatin. J. Int. Med. Res. *36*, 1273–1278.

38. Guan, Y.S., Liu, Y., Zou, Q., He, Q., La, Z., Yang, L., and Hu, Y. (2009). Adenovirus-mediated wild-type p53 gene transfer in combination with bronchial arterial infusion for treatment of advanced non-small-cell lung cancer, one year follow-up. J. Zhejiang Univ. Sci. B *10*, 331–340.

39. Pan, J.J., Zhang, S.W., Chen, C.B., Xiao, S.W., Sun, Y., Liu, C.Q., Su, X., Li, D.M., Xu, G., Xu, B., and Lu, Y.Y. (2009). Effect of recombinant adenovirus-p53 combined with radiotherapy on long-term prognosis of advanced nasopharyngeal carcinoma. J. Clin. Oncol. *27*, 799–804.

40. Tian, G., Liu, J., Zhou, J.S.R., and Chen, W. (2009). Multiple hepatic arterial injections of recombinant adenovirus p53 and 5-fluorouracil after transcatheter arterial chemoembolization for unresectable hepatocellular carcinoma: a pilot phase II trial. Anticancer Drugs *20*, 389–395.

41. Yang, Z.X., Wang, D., Wang, G., Zhang, Q.H., Liu, J.M., Peng, P., and Liu, X.H. (2010). Clinical study of recombinant adenovirus-p53 combined with fractionated stereotactic radiotherapy for hepatocellular carcinoma. J. Cancer Res. Clin. Oncol. *136*, 625–630.

42. Heo, J., Breitbach, C.J., Moon, A., Kim, C.W., Patt, R., Kim, M.K., Lee, Y.K., Oh, S.Y., Woo, H.Y., Parato, K., et al. (2011). Sequential therapy with JX-594, a targeted oncolytic poxvirus, followed by sorafenib in hepatocellular carcinoma: Preclinical and clinical demonstration of combination efficacy. Mol. Ther. *19*, 1170–1179.

43. Cerullo, V., Diaconu, I., Kangasniemi, L., Rajecki, M., Escutenaire, S., Koski, A., Romano, V., Rouvinen, N., Tuuminen, T., Laasonen, L., et al. (2011). Immunological effects of low-dose cyclophosphamide in cancer patients treated with oncolytic adenovirus. Mol. Ther. *19*, 1737–1746.

44. Koski, A., Raki, M., Nokisalmi, P., Liikanen, I., Kangasniemi, L., Joensuu, T., Kanerva, A., Pesonen, S., Alemany, R., and Hemminki, A. (2012). Verapamil results in increased blood levels of oncolytic adenovirus in treatment of patients with advanced cancer. Mol. Ther. *20*, 221–229.

45. Suriano, R., Rajoria, S., George, A.L., Geliebter, J., Tiwari, R.K., and Wallack, M. (2013). Follow-up analysis of a randomized phase III immunotherapeutic clinical trial on melanoma. Mol. Clin. Oncol. *1*, 466–472.

46. Westphal, M., Ylä-Herttuala, S., Martin, J., Warnke, P., Menei, P., Eckland, D., Kinley, J., Kay, R., and Ram, Z.; ASPECT Study Group (2013). Adenovirus-mediated gene therapy with sitimagene ceradenovec followed by intravenous ganciclovir for patients with operable high-grade glioma (ASPECT): a randomised, open-label, phase 3 trial. Lancet Oncol. *14*, 823–833.

47. Dong, J., Li, W., Dong, A., Mao, S., Shen, L., Li, S., Gong, X., and Wu, P. (2014). Gene therapy for unresectable hepatocellular carcinoma using recombinant human adenovirus type 5. Med. Oncol. *31*, 95.

48. Freytag, S.O., Stricker, H., Lu, M., Elshaikh, M., Aref, I., Pradhan, D., Levin, K., Kim, J.H., Peabody, J., Siddiqui, F., et al. (2014). Prospective randomized phase 2 trial of intensity modulated radiation therapy with or without oncolytic adenovirus-mediated cytotoxic gene therapy in intermediate-risk prostate cancer. Int. J. Radiat. Oncol. Biol. Phys. *89*, 268–276.

49. Doniņa, S., Strēle, I., Proboka, G., Auziņš, J., Alberts, P., Jonsson, B., Venskus, D., and Muceniece, A. (2015). Adapted ECHO-7 virus Rigvir immunotherapy (oncolytic virotherapy) prolongs survival in melanoma patients after surgical excision of the tumour in a retrospective study. Melanoma Res. *25*, 421–426.

50. Kanerva, A., Koski, A., Liikanen, I., Oksanen, M., Joensuu, T., Hemminki, O., Palmgren, J., Hemminki, K., and Hemminki, A. (2015). Case-control estimation of the impact of oncolytic adenovirus on the survival of patients with refractory solid tumors. Mol. Ther. *23*, 321–329.

51. Lin, X.J., Li, Q.J., Lao, X.M., Yang, H., and Li, S.P. (2015). Transarterial injection of recombinant human type-5 adenovirus H101 in combination with transarterial chemoembolization (TACE) improves overall and progressive-free survival in unresectable hepatocellular carcinoma (HCC). BMC Cancer *15*, 707.

52. Andtbacka, R.H.I., Ross, M., Puzanov, I., Milhem, M., Collichio, F., Delman, K.A., Amatruda, T., Zager, J.S., Cranmer, L., Hsueh, E., et al. (2016). Patterns of clinical response with talimogene laherparepvec (T-VEC) in patients with melanoma treated in the OPTiM phase III clinical trial. Ann. Surg. Oncol. *23*, 4169–4177.

53. Gao, Y.D., Chen, G.L., Guo, P.D., and Yang, X.L. (2016). [Study on the application effect of Once Vex^{GM-CSF} and adriamycin in the interventional therapy of malignant tumor in clinical curative effect]. Zhonghua Yi Xue Za Zhi *96*, 2173–2175.

54. Su, X., Chen, W.J., Xiao, S.W., Li, X.F., Xu, G., Pan, J.J., and Zhang, S.W. (2016). Effect and safety of recombinant adenovirus-*p53* transfer combined with radiotherapy on long-term survival of locally advanced cervical cancer. Hum. Gene Ther. *27*, 1008–1014.

55. Harrington, K.J., Andtbacka, R.H., Collichio, F., Downey, G., Chen, L., Szabo, Z., and Kaufman, H.L. (2016). Efficacy and safety of talimogene laherparepvec versus granulocyte-macrophage colony-stimulating factor in patients with stage IIIB/C and IVM1a melanoma: Subanalysis of the phase III OPTiM trial. OncoTargets Ther. *9*, 7081–7093.

56. Ma, W.-S., Ma, J.-G., and Xing, L.-N. (2017). Efficacy and safety of recombinant human adenovirus p53 combined with chemoradiotherapy in the treatment of recurrent nasopharyngeal carcinoma. Anticancer Drugs *28*, 230–236.

57. Cohn, D.E., Sill, M.W., Walker, J.L., O'Malley, D., Nagel, C.I., Rutledge, T.L., Bradley, W., Richardson, D.L., Moxley, K.M., and Aghajanian, C. (2017). Randomized phase IIB evaluation of weekly paclitaxel versus weekly paclitaxel with oncolytic reovirus (Reolysin®) in recurrent ovarian, tubal, or peritoneal cancer: An NRG Oncology/Gynecologic Oncology Group study. Gynecol. Oncol. *146*, 477–483.

58. Bradbury, P.A., Morris, D.G., Nicholas, G., Tu, D., Tehfe, M., Goffin, J.R., Shepherd, F.A., Gregg, R.W., Rothenstein, J., Lee, C., et al. (2018). Canadian Cancer Trials Group (CCTG) IND211: A randomized trial of pelareorep (Reolysin) in patients with previously treated advanced or metastatic non-small cell lung cancer receiving standard salvage therapy. Lung Cancer *120*, 142–148.

59. Liu, J., Lv, D., Wang, H., Zou, J., Chen, F., and Yang, H. (2018). Recombinant adenovirus-p53 enhances the therapeutic effect of surgery and chemoradiotherapy combination in hypopharyngeal squamous cell carcinomas patients. Medicine (Baltimore) *97*, e12193.

60. He, C., Zhang, Y., and Lin, X. (2018). Increased overall survival and decreased cancer-specific mortality in patients with hepatocellular carcinoma treated by transarterial chemoembolization and human adenovirus type-5 combination therapy: A competing risk analysis. J. Gastrointest. Surg. *22*, 989–997.

61. Schenk, E.L., Mandrekar, S.J., Dy, G.K., Aubry, M.C., Tan, A.D., Dakhil, S.R., Sachs, B.A., Nieva, J.J., Bertino, E., Lee Hann, C., et al. (2020). A randomized double-blind phase II study of the Seneca Valley virus (NTX-010) versus placebo for patients with extensive-stage SCLC (ES SCLC) who were stable or responding after at least four cycles of platinum-based chemotherapy: North Central Cancer Treatment Group (Alliance) N0923 Study. J. Thorac. Oncol. *15*, 110–119.

62. Bommareddy, P.K., Patel, A., Hossain, S., and Kaufman, H.L. (2017). Talimogene laherparepvec (T-VEC) and other oncolytic viruses for the treatment of melanoma. Am. J. Clin. Dermatol. *18*, 1–15.

63. Andtbacka, R.H.I., Kaufman, H.L., Collichio, F., Amatruda, T., Senzer, N., Chesney, J., Delman, K.A., Spitler, L.E., Puzanov, I., Agarwala, S.S., et al. (2015). Talimogene laherparepvec improves durable response rate in patients with advanced melanoma. J. Clin. Oncol. *33*, 2780–2788.

64. Zhang, Q.-N., Li, Y., Zhao, Q., Tian, M., Chen, L.-L., Miao, L.-Y., and Zhou, Y.-J. (2021). Recombinant human adenovirus type 5 (Oncorine) reverses resistance to immune checkpoint inhibitor in a patient with recurrent non-small cell lung cancer: A case report. Thorac. Cancer *12*, 1617–1619.

65. Liang, M. (2018). Oncorine, the world first oncolytic virus medicine and its update in China. Curr. Cancer Drug Targets *18*, 171–176.

66. Peter, M., and Kühnel, F. (2020). Oncolytic adenovirus in cancer immunotherapy. Cancers (Basel) *12*, 3354.

67. Aref, S., Bailey, K., and Fielding, A. (2016). Measles to the rescue: A review of oncolytic measles virus. Viruses *8*, 294.

68. Müller, L., Berkeley, R., Barr, T., Ilett, E., and Errington-Mais, F. (2020). Past, present and future of oncolytic reovirus. Cancers (Basel) *12*, 3219.

69. Basler, M., and Groettrup, M. (2007). Advances in prostate cancer immunotherapies. Drugs Aging *24*, 197–221.

70. Stojdl, D.F., Lichty, B., Knowles, S., Marius, R., Atkins, H., Sonenberg, N., and Bell, J.C. (2000). Exploiting tumor-specific defects in the interferon pathway with a previously unknown oncolytic virus. Nat. Med. *6*, 821–825.

71. Mahalingam, D., Fountzilas, C., Moseley, J., Noronha, N., Tran, H., Chakrabarty, R., Selvaggi, G., Coffey, M., Thompson, B., and Sarantopoulos, J. (2017). A phase II study of REOLYSIN® (pelareorep) in combination with carboplatin and paclitaxel for patients with advanced malignant melanoma. Cancer Chemother. Pharmacol. *79*, 697–703.

72. Mahalingam, D., Wilkinson, G.A., Eng, K.H., Fields, P., Raber, P., Moseley, J.L., Cheetham, K., Coffey, M., Nuovo, G., Kalinski, P., et al. (2020). Pembrolizumab in combination with the oncolytic virus pelareorep and chemotherapy in patients with advanced pancreatic adenocarcinoma: A phase Ib study. Clin. Cancer Res. *26*, 71–81.

73. Puzanov, I., Milhem, M.M., Minor, D., Hamid, O., Li, A., Chen, L., Chastain, M., Gorski, K.S., Anderson, A., Chou, J., et al. (2016). Talimogene laherparepvec in combination with ipilimumab in previously untreated, unresectable stage IIIB–IV melanoma. J. Clin. Oncol. *34*, 2619–2626.

74. Rommelfanger, D.M., Compte, M., Diaz, R.M., Ilett, E., Alvarez-Vallina, L., Thompson, J.M., Kottke, T.J., Melcher, A., and Vile, R.G. (2013). The efficacy versus toxicity profile of combination virotherapy and TLR immunotherapy highlights the danger of administering TLR agonists to oncolytic virus-treated mice. Mol. Ther. *21*, 348–357.

75. Packer, R.J., Raffel, C., Villablanca, J.G., Tonn, J.C., Burdach, S.E., Burger, K., LaFond, D., McComb, J.G., Cogen, P.H., Vezina, G., and Kapcala, L.P. (2000). Treatment of progressive or recurrent pediatric malignant supratentorial brain tumors with herpes simplex virus thymidine kinase gene vector-producer cells followed by intravenous ganciclovir administration. J. Neurosurg. *92*, 249–254.

76. Burke, M.J., Ahern, C., Weigel, B.J., Poirier, J.T., Rudin, C.M., Chen, Y., Cripe, T.P., Bernhardt, M.B., and Blaney, S.M. (2015). Phase I trial of Seneca Valley virus (NTX-010) in children with relapsed/refractory solid tumors: a report of the Children's Oncology Group. Pediatr. Blood Cancer *62*, 743–750.

77. Kolb, E.A., Sampson, V., Stabley, D., Walter, A., Sol-Church, K., Cripe, T., Hingorani, P., Ahern, C.H., Weigel, B.J., Zwiebel, J., and Blaney, S.M. (2015). A phase I trial and viral clearance study of reovirus (Reolysin) in children with relapsed or refractory extra-cranial solid tumors: a Children's Oncology Group Phase I Consortium report. Pediatr. Blood Cancer *62*, 751–758.

78. Li, L., Liu, S., Han, D., Tang, B., and Ma, J. (2020). Delivery and biosafety of oncolytic virotherapy. Front. Oncol. *10*, 475.

79. Power, A.T., Wang, J., Falls, T.J., Paterson, J.M., Parato, K.A., Lichty, B.D., Stojdl, D.F., Forsyth, P.A.J., Atkins, H., and Bell, J.C. (2007). Carrier cell-based delivery of an oncolytic virus circumvents antiviral immunity. Mol. Ther. *15*, 123–130.

80. Evgin, L., Acuna, S.A., Tanese de Souza, C., Marguerie, M., Lemay, C.G., Ilkow, C.S., Findlay, C.S., Falls, T., Parato, K.A., Hanwell, D., et al. (2015). Complement inhibition prevents oncolytic vaccinia virus neutralization in immune humans and cynomolgus macaques. Mol. Ther. *23*, 1066–1076.

81. Evgin, L., Ilkow, C.S., Bourgeois-Daigneault, M.-C., de Souza, C.T., Stubbert, L., Huh, M.S., Jennings, V.A., Marguerie, M., Acuna, S.A., Keller, B.A., et al. (2016). Complement inhibition enables tumor delivery of LCMV glycoprotein pseudotyped viruses in the presence of antiviral antibodies. Mol. Ther. Oncolytics *3*, 16027.

82. Marotel, M., Hasim, M.S., Hagerman, A., and Ardolino, M. (2020). The two-faces of NK cells in oncolytic virotherapy. Cytokine Growth Factor Rev. *56*, 59–68.

83. Ungerechts, G., Bossow, S., Leuchs, B., Holm, P.S., Rommelaere, J., Coffey, M., Coffin, R., Bell, J., and Nettelbeck, D.M. (2016). Moving oncolytic viruses into the clinic: Clinical-grade production, purification, and characterization of diverse oncolytic viruses. Mol. Ther. Methods Clin. Dev. *3*, 16018.

84. Working, P.K., Lin, A., and Borellini, F. (2005). Meeting product development challenges in manufacturing clinical grade oncolytic adenoviruses. Oncogene *24*, 7792–7801.

85. Yamaguchi, T., and Uchida, E. (2018). Oncolytic virus: Regulatory aspects from quality control to clinical studies. Curr. Cancer Drug Targets *18*, 202–208.

86. Almutairi, A.R., Alkhatib, N.S., Oh, M., Curiel-Lewandrowski, C., Babiker, H.M., Cranmer, L.D., McBride, A., and Abraham, I. (2019). Economic evaluation of talimogene laherparepvec plus ipilimumab combination therapy vs ipilimumab monotherapy in patients with advanced unresectable melanoma. JAMA Dermatol. *155*, 22–28.

87. Corrigan, P.A., Beaulieu, C., Patel, R.B., and Lowe, D.K. (2017). Talimogene laherparepvec: An oncolytic virus therapy for melanoma. Ann. Pharmacother. *51*, 675–681.

88. Inno, A., Lo Russo, G., Salgarello, M., Corrao, G., Casolino, R., Galli, G., Modena, A., Romano, L., Pusceddu, S., Greco, F.G., et al. (2018). The evolving landscape of criteria for evaluating tumor response in the era of cancer immunotherapy: From Karnofsky to iRECIST. Tumori *104*, 88–95.

89. McNamara, M.G., Jacobs, T., Lamarca, A., Hubner, R.A., Valle, J.W., and Amir, E. (2020). Impact of high tumor mutational burden in solid tumors and challenges for biomarker application. Cancer Treat. Rev. *89*, 102084.

90. Wickham, H. (2009). ggplot2 (Springer).

OMTO, Volume *23*

Supplemental information

A systematic analysis on the clinical safety and efficacy of onco-virotherapy

Darshak K. Bhatt, Lieske Wekema, Luciana Rodrigues Carvalho Barros, Roger Chammas, and Toos Daemen

Supplementary Information

1. Search Strategy

We retrieved clinical trials from the *clinicaltrials.gov* registry (https://clinicaltrials.gov) and PubMed (http://www.ncbi.nlm.nih.gov/PubMed) and EMBASE (https://www.embase.com) databases at the start of August 2020. For each medium we used a different search strategy, as specified below.

- *ClinicalTrials.gov*

We obtained clinical trials from the *clinicaltrials.gov* registry using the following settings:

Status: “All studies” / Condition or disease: “Cancer” / Other terms: “Virus”

- *PubMed*

In cooperation with the Central Medical Library (CMB) of the University Medical Center Groningen (UMCG), we constructed a PubMed search strategy based on our PICOS framework:

("Neoplasms"[Mesh] OR "Oncolytic Virotherapy"[Mesh] OR neoplasia*[tiab] OR neoplasm*[tiab] OR tumor[tiab] OR tumors[tiab] OR tumour*[tiab] OR cancer*[tiab] OR malignan*[tiab] OR carcinoma*[tiab] OR metasta*[tiab] OR melanom*[tiab] OR oncolyt*[tiab])

AND

("Oncolytic Virotherapy"[Mesh] OR "Oncolytic Viruses"[Mesh] OR virotherap*[tiab] OR (oncoly*[tiab] AND (virus*[tiab] OR viral[tiab] OR adenovir*[tiab] OR reovirus*[tiab] OR poxvirus*[tiab] OR gene[tiab] OR genes[tiab] OR herpes*[tiab])) OR ((administration*[ti] OR target*[ti] OR therap*[ti] OR treatment[ti] OR intratumo*[tiab] OR inject*[ti] OR intravenous[ti] OR immunotherap*[ti]) AND (virus*[ti] OR reolysin[ti] OR reovirus*[ti] OR poxvirus[ti] OR adenovirus*[ti])) OR replicon*[tiab])

AND

("Clinical Trial" [Publication Type] OR "Clinical Trials as Topic"[Mesh] OR "Cohort Studies"[Mesh] OR "Observational Study" [Publication Type] OR "Case Reports" [Publication Type] OR "Prognosis"[Mesh] OR random*[tiab] OR controlled study[tiab] OR clinical study[tiab] OR trial[tiab] OR cohort[tiab] OR prospectiv*[tiab] OR follow-up[tiab] OR study[ti])

NOT

("Animals"[Mesh] NOT "Humans"[Mesh])

NOT

(epstein[tiab] OR epstein[ti] OR EBV[tiab] OR papillomavir*[tiab] OR papillomavir*[ti] OR hepatitis*[tiab] OR hepatitis[ti] OR HCV[tiab] OR HCV[ti] OR HBV[ti] OR HBV[tiab] OR HIV[tiab] OR HIV[ti])

- *EMBASE*

In cooperation with the CMB of the UMCG, we re-wrote the PubMed search strategy into a search strategy that was suitable for the EMBASE database:

('neoplasm'/exp OR 'oncolytic virotherapy'/exp OR (neoplasia* OR neoplasm* OR tumor OR tumors OR tumour* OR cancer* OR malignan* OR carcinoma* OR metasta* OR melanom* OR oncolyt*):ab,ti)

AND

('oncolytic virotherapy'/exp OR 'oncolytic virus'/exp OR virotherap*:ab,ti OR (oncoly* AND (virus* OR viral OR adenovir* OR reovirus* OR poxvirus* OR gene OR genes OR herpes*)):ab,ti OR ((administration* OR target* OR therap* OR treatment OR intratumo*

OR inject* OR intravenous OR immunotherap* OR trial) AND (virus* OR reolysin OR reovirus* OR poxvirus OR adenovirus*)):ti OR replicon*:ab,ti)

AND

('clinical trial'/exp OR 'intervention study'/exp OR 'longitudinal study'/exp OR 'major clinical study'/exp OR 'prospective study'/exp OR 'cohort analysis'/exp OR 'follow up'/exp OR 'observational study'/exp OR (random* OR 'controlled study' OR 'clinical study' OR trial OR cohort OR prospectiv* OR 'follow-up'):ab,ti OR study:ti)

NOT

('animal'/exp NOT 'human'/exp)

NOT

('conference abstract'/it OR 'review'/it)

NOT

(hepatitis:ti OR hiv:ti OR 'epstein barr':ti)

2. **Supplementary table 1, containing the dataset obtained after systematic search and review; with each article or trial listed in row, while respective variable of interest organized in columns.**
